# Association of the derived neutrophil-to-lymphocyte ratio with cardiovascular and all-cause mortality

**DOI:** 10.1371/journal.pone.0324849

**Published:** 2025-06-05

**Authors:** Jun Ma, Yingda Song, Shaochen Zhang, Weirong Feng, Ziqing Wei, Zengqiang Shen, Zhiqiang Tong, Xiaoming Bai

**Affiliations:** 1 The Thoracic Surgery Department of Shanxi Provincial People’s Hospital, Shanxi Medical University, Taiyuan, Shanxi Province, China; 2 Second Clinical Medical College, Shanxi Medical University, Taiyuan, Shanxi Province, China; Universitas Indonesia Fakultas Kedokteran, INDONESIA

## Abstract

**Purpose:**

Accumulating evidence supports the important role of inflammation in disease outcomes. Derived neutrophil-to-lymphocyte ratio (dNLR) is broadly identified as potential prognostic marker in clinical trials or daily clinical practice, but dNLR has never been verified in cardiovascular and all-cause mortality.

**Methods:**

Overall, 34,392 participants from the National Health and Nutrition Examination Survey (NHANES) were included. The exposure variable was Log-dNLR (dNLR Logarithmic transformation). Participants were categorized according to Log-dNLR quartiles and followed through 31st December 2019. Weighted univariable and multivariable Cox regression were applied to assess the relationship between Log-dNLR evaluated as categorical variables, with cardiovascular and all-cause mortality. Restricted cubic spline (RCS) regression, subgroup analysis, and threshold effect were applied to assess nonlinear relationship between Log-dNLR with cardiovascular and all-cause mortality as well as the effects of special populations. We used multiple sensitivity analyses to reduce selection bias and validate these relationships. Last, the time-dependent weighted receiver operating characteristic (ROC) curve analysis was used to assess predictive accuracy of the Log-dNLR for survival outcomes.

**Results:**

During a median follow-up duration of 116.90 months, a total of 4,939 all-cause deaths occurred, of which 1,327 were cardiovascular deaths. After adjusting for multiple confounders, compared to the quartile 1, the hazard ratios (HRs) (95% confidence interval, CI) for quartile 4 were 1.38 (95% CI, 1.14–1.68) for cardiovascular mortality and 1.16 (95% CI, 1.06–1.27) for all-cause mortality was identified using RCS regression, with a threshold point of 0.370. Significant differences were observed before and after this threshold point. In addition, there were significant interactions between sex, hypertension status and Log-dNLR (*P* for interaction = 0.025, 0.005, respectively) for the all-cause mortality risk and significant interactions between age groups, diabetes status and Log-dNLR (*P* for interaction = 0.007, 0.004, respectively) for the cardiovascular mortality risk. Lastly, ROC analysis revealed that Log-dNLR showed moderate predictive power for all-cause and cardiovascular mortality in the short and long term.

**Conclusions:**

In summary, elevated dNLR levels are significantly associated with an increased risk of both cardiovascular and all-cause mortality.

## Introduction

Cardiovascular disease remains the leading cause of death worldwide and also continues to be a significant public health challenge on morbidity, quality of life, and societal costs throughout the world [1,2]. It is defined by the presence of both the heart and blood vessels diseases which encompasses coronary heart or artery disease, peripheral artery disease, and acute coronary syndrome [3]. Previous studies have implicated that systemic inflammation is a significant associated factor in the onset, progression, and clinical outcome of cardiovascular disease. Some blood routine indexes such as C-reactive protein [4], neutrophils [5], and monocytes [6] which represent systemic inflammation, have been reported to be implicated in cardiovascular disease. Moreover, a healthy lifestyle [7] and the Mediterranean diet [8] could reduce and prevent cardiovascular event risk. Consequently, the prevention and management of cardiovascular risk factors are of paramount importance.

A novel biomarker, Log-dNLR (Logarithmic transformated derived neutrophil-to-lymphocyte ratio) has been of intense interest and has been recognized as a predictor that participates in chronic inflammatory diseases and cancers. Some evidence suggests that the elevated dNLR levels have been associated with higher incidence of many medical conditions that affect mortality risk, including limb ischemia [9], acute coronary syndrome [10], cancers [11,12], and infection of COVID-19 [13]. A elevated dNLR level is indicative of a notably poor prognosis and heightened incidence across a range of diseases, suggesting its potential as a prognostic marker. Nevertheless, there are limited investigation on the association of dNLR indices with cardiovascular and all-cause mortality.

Hence we hypothesized that dNLR is associated with cardiovascular and all-cause mortality. In the present study, we aim to investigate the associations between the dNLR with cardiovascular mortality and all-cause mortality in the National Health and Nutrition Examination Survey (NHANES), thereby extending the epidemiological understanding of dNLR and assessing its clinical implications for mortality outcomes.

## Methods

### Data source and study population

The NHANES is a cross-sectional survey in the United States. Survey of relevant interview, examination, dietary, and laboratory data were collected from adults and children of the United States population through method of “stratified multistage probability sampling”. The United States of America (US) National Center for Health Statistics (NCHS) Research Ethics Review Board approved all studies of the NHANES protocol, and each participant signed written informed consent. All data from the NHANES database have undergone ethical approval statement that approved by the National center for health statistics Ethics Review board approval, for details please see the NHANES website (https://www.cdc.gov/nchs/nhanes/index.htm).

**Fig 1:**
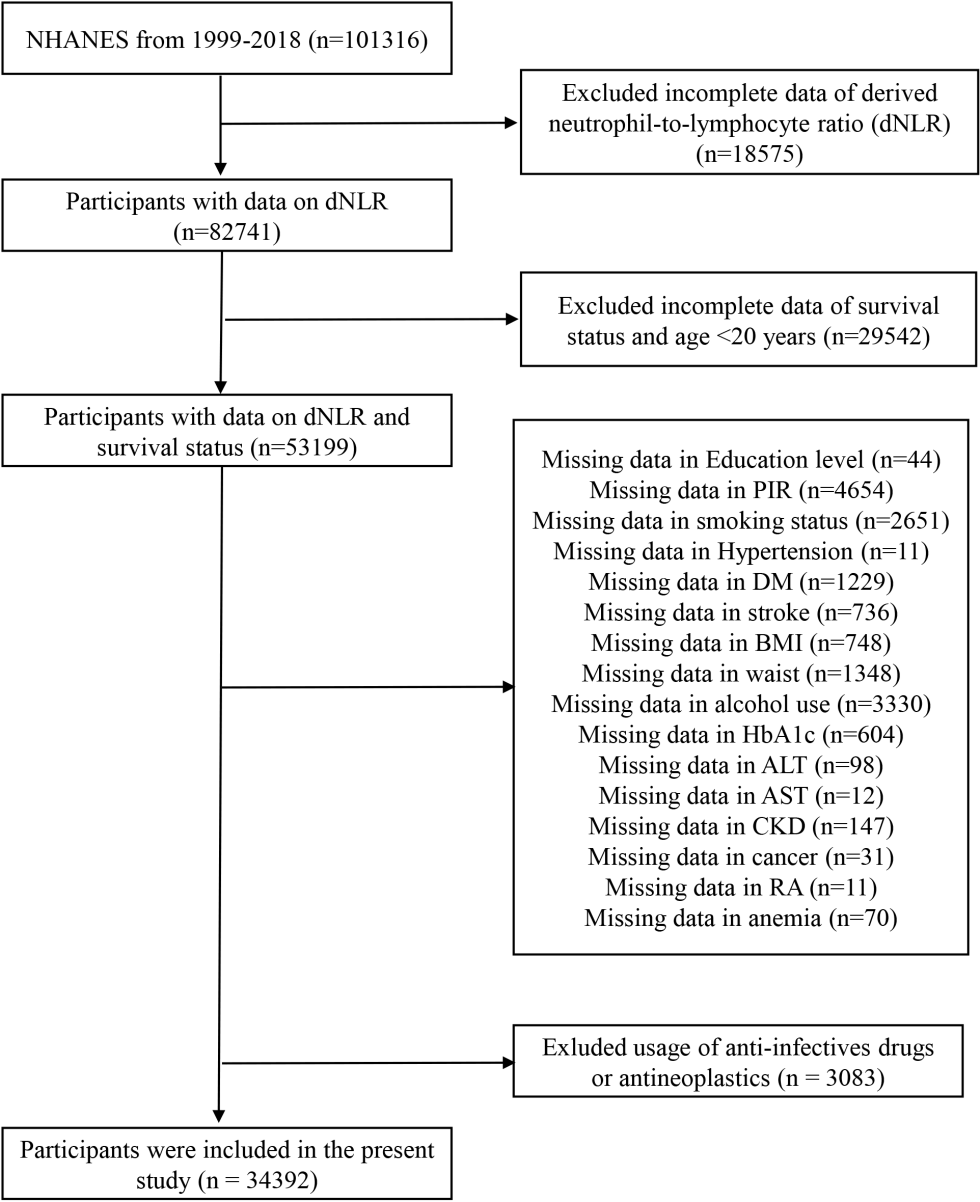
Flow diagram.

Initially, a total of 34,392 participants were chosen from cohorts of 1999−2018 consecutive NHANES cycles. Thereafter, the exclusion criteria were: age of participants was restricted in ≥ 20 years; participants without blood routine examination data; participants without follow-up information; participants without complete covariate information; usage of anti-infectives drugs or antineoplastics in participants (Fig 1). Given the low proportion of missing data, we opted for listwise deletion rather than performing multiple imputation.

### Measurement of the dNLR

The dNLR is a systemic inflammation marker that reflects the balance between neutrophils and lymphocytes in peripheral blood. It is calculated as neutrophil count/ (white blood cell count – neutrophil count) (expressed as × 10^3^ cells/μL) per previous publications [14]. This formula is based on the traditional neutrophil-to-lymphocyte ratio (NLR), but dNLR is considered a more accessible alternative when absolute lymphocyte counts are not directly available. The serum samples of participants were measured with the Beckman Coulter MAXM instrument in the Mobile Examination Center (MEC). Detailed measurement methods of blood collection and processing are provided in the NHANES Laboratory Procedure Manual.

### Outcome ascertainment

Follow-up and mortality data of the NHANES were obtained from the National Death Index (NDI) of NCHS. In the study, the follow-up time of participants was followed through the death date or the last follow-up date (December 31, 2019). The causes and cases of death were identified via active follow-up survey, physicians reviewing medical records or death certificates. The primary outcome comprised final mortality status, follow-up time, and the leading causes of mortality. All-cause mortality was ascertained as death due to any cause during the follow-up time; cardiovascular mortality was ascertained as death due to codes I00-I09, I11, I13, I20-I51, or I60-I69, according to the International Classification of Diseases, 10th revision (ICD-10).

### Covariates

These prior covariates that could as potential confounders of the associations between dNLR with cardiovascular and all-cause mortality were chosen based on the existing literature and clinical consideration. For each individual, the demographic and lifestyle information such as age, sex, race, educational level, poverty index ratio (PIR), alcohol use, and smoking status was acquired from the standardized household questionnaire. Age was categorized as four age groups (20−39 years, 40−59 years, 60−79 years, and ≥ 80 years), with the 20−39 years group as the reference; race was coded as non-Hispanic white, non-Hispanic black (as the reference), Mexican American, and others; Education level was graded into primary school and less (as the reference), middle and high school, and college and higher; Socioeconomic level was divided the individuals into PIR ≤ 1.0 group (as the reference) and PIR > 1.0 group based on the federal poverty line (PIR = 1). The following laboratory indicators were obtained from the examination data according to standard protocols: body mass index (BMI), waist circumference, albumin, alanine aminotransferase (ALT), aspartate aminotransferase (AST), HbA1c, and serum creatinine. In addition, history of present comorbidities including diabetes (DM), prediabetes (pre-DM), cardiovascular disease (CVD), hypertension, hyperlipidemia, cancer status, chronic kidney disease (CKD), stroke, rheumatoid arthritis (RA), anemia, alcohol use, and smoking status were considered as potential determinants of mortality. The diagnostic criteria for DM are: (1) diagnosed with diabetes by doctors; (2) glycohemoglobin HbA1c > 6.5%; (3) fasting glucose ≥ 7.0 mmol/l; (4) random blood glucose ≥ 11.1 mmol/l; (5) two-hour OGTT blood glucose ≥ 11.1 mmol/l; (6) use of diabetes medication or insulin. The diagnostic criteria of pre-DM are: (1) diagnosed with prediabetes by doctors; (2) glycohemoglobin HbA1c 5.7% − 6.5%; (3) fasting glucose 5.6 mmol/l – 7.0 mmol/l; (4) two-hour OGTT blood glucose 7.8 mmol/l – 11.1 mmol/l. The diagnostic criteria of hypertension are: (1) average systolic blood pressure ≥ 140 mmHg or average diastolic blood pressure ≥ 90 mmHg at least three times; (2) use of antihypertensive drug; (3) subjects or doctor-reported hypertension diagnosis. The diagnostic criteria for hyperlipidemia are: (1) elevated total cholesterol ≥ 5.18 mmol/L; (2) triglyceride level ≥ 150 mg/dL; (3) high density lipoprotein-cholesterol < 1.04 mmol/L in male and 1.30 mmol/L in female; (4) low density lipoprotein-cholesterol ≥ 3.37 mmol/L; (5) use of antihyperlipidemic drugs. Cardiovascular disease was an aggregate of congestive heart failure, coronary heart disease, heart attack, angina, and stroke. The diagnostic criteria for congestive heart failure was based on personal questionnaire surveys of “has a doctor or other health professional ever told you had congestive heart failure (mcq160b)?” The diagnostic criteria for coronary heart disease was based on personal questionnaire surveys of “has a doctor or other health professional ever told you had coronary heart disease (mcq160c)?” The diagnostic criteria for heart attack were based on personal questionnaire surveys of “has a doctor or other health professional ever told you had heart attack or myocardial infarction (mcq160e)?” and “has a doctor or other health professional ever told you had heart attack (spq070e)?” The diagnostic criteria for angina were based on personal questionnaire surveys of “has a doctor or other health professional ever told you had angina or angina pectoris (mcq160d)?” The diagnostic criteria for stroke were ascertained by personal questionnaire surveys of “has a doctor or other health professional ever told you had stroke (mcq160f)?” and “has a doctor or other health professional ever told you had stroke (spd070d)?” The diagnostic criteria for cancer status was evaluated by the medical status questionnaire of “has a doctor or other health professional ever told you had cancer or malignancy (mcq220)?” Albumin to creatinine ratio (ACR) ≥ 30 mg/g (3 mg/mmol) and/or estimated Glomerular Filtration Rate (eGFR) < 60 mL/min/ 1.73m^2^ were used as definition of CKD according to the KDIGO 2021 Guideline. The diagnostic criteria for RA were based on questionnaire surveys of “doctor ever said you had arthritis (mcq160a)?” and “which type of arthritis was it (mcq190)?” Based on the World Health Organization (WHO) criteria, anemia was defined as Hb < 12 g/dL and < 13 g/dL in women and men, respectively. Alcohol use was categorized as former (had ≥ 12 drinks in 1 year and did not drink last year, or did not drink last year but drank ≥12 drinks in lifetime), never (had < 12 drinks in lifetime), mild (1 drinks per day for female and 2 drinks per day for male), moderate (≥ 2 drinks per day for for female and ≥ 3 drinks per day for for male, or binge drinking ≥ 2 days per month), or heavy (≥ 3 drinks per day for for female and ≥ 4 drinks per day for for male, or binge drinking ≥ 5 days per month). Individual smoking more than 100 cigarettes in life and smoking some days or every day was defined as smokers.

### Statistical analysis

NHANES employs a complex, multistage probability sampling design, requiring the use of survey weights to obtain nationally representative estimates. In this study, we used examination weights (WTMEC2YR/WTMEC4YR), as our primary exposure variable (dNLR) was derived from laboratory data. When combining multiple NHANES cycles, weights were adjusted according to NHANES analytic guidelines to account for varying cycle lengths. Data from each study subject were weighted to correct the complexity of the sampling survey.

For the baseline characteristics, continuous variables were described as mean and standard deviation (SD), and categorical variables were summarized as frequency and percentage. Comparisons of baseline characteristics for categorical variables were compared using the chi-square test, and the t-test or one-way analysis of variance for continuous variables. Moreover, for the non-normal distribution of dNLR data, we used Logarithmic transformations to obtain normal distributions of data (named Log-dNLR) by validation of Kolmogorov-Smirnov test. The Log-dNLR were categorized into quartiles as follows: quartile 1 (0.003–0.319); quartile 2 (0.319–0.380); quartile 3 (0.380–0.446), and quartile 4 (0.446–1.501). We calculated cut-off values and evaluated sensitivity and specificity between dNLR with cardiovascular or all-cause mortality by receiver operating characteristic (ROC) curve. Univariable Cox regression analysis was first conducted to select covariates with *P* ≤ 0.05; multivariable Cox regression models including crude models to model 3 were used for estimating the hazard ratios (HR) and corresponding 95% confidence intervals in Log-dNLR as a continuous variable and Log-dNLR quartiles as a categorical variable. The test of trend was evaluated by assigning the median value of each quartile of Log-dNLR and entering it as a continuous variable. In multivariable Cox regression analysis of all-cause mortality, the crude model was not adjusted for any covariates; model 1 was adjusted for age, sex, PIR, race, and education; model 2 was further adjusted for BMI, waist circumference, albumin, AST, HbA1c (%), and serum creatinine; model 3 was adjusted for the variables in model 2 and additional confounders, including DM status, CVD, hypertension, hyperlipidemia, cancer status, CKD, stroke, alcohol use, RA, anemia, and smoking status. In multivariable Cox regression analysis of cardiovascular mortality (compared with all-cause mortality), model 2 was adjusted for age, sex, PIR, race, education, BMI, waist circumference, albumin, ALT, HbA1c, and serum creatinine. The subgroup analysis was used to assess the heterogeneity between the Log-dNLR with cardiovascular and all-cause mortality in the distinct populations, including the following variables of age groups, PIR, sex, race, education, DM status, CVD, hypertension, hyperlipidemia, CKD, stroke, cancer status, alcohol use, RA, anemia, and smoking status. RCS analysis was used to flexibly model the trends between Log-dNLR and cardiovascular, all-cause mortality under the full covariates adjustment. The threshold effect was calculated using a segmented regression to fit the two-piecewise linear association between Log-dNLR and cardiovascular, all-cause mortality risk. We tested for potential non-linearity based on a likelihood ratio test comparing the linear regression model with the Restricted cubic spline (RCS) model. In addition, special populations were researched in cardiovascular, all-cause mortality by RCS analysis. Multiple sensitivity analysis was also conducted to evaluate the robustness of the results, including removing participants with CVD or cancer at baseline, removing individuals who died within 24 months of follow-up, and removing participants with the shortest follow-up duration (NHANES 2017–2018). Finally, to assess the effectiveness of the Log-dNLR as a predictor of survival outcomes, we employed time-dependent weighted ROC curves. R statistical software (version 4.2.2) including packages “nhanesR”, “rms”, “pROC”, and “survey” was used for all statistical analyses and plotting. The significance threshold of *P* value was set at < 0.05, and all analyses of *P* values were two-sided.

## Results

### Baseline characteristics

We included 34,392 US individuals aged 20 years and older from NHANES (1999-2018). During a mean follow-up of 116.90 ± 1.05 months (mean ± SD), a total of 4,939 all-cause deaths occurred, of which 1,327 were cardiovascular deaths. Table 1 showed the baseline characteristics of participants grouped according to Log-dNLR quartiles. Participants with higher Log-dNLR had a higher prevalence of DM, CVD, hypertension, hyperlipidemia, cancer status, CKD, and stroke (Table 1). Participants in the all-cause mortality group were older, more likely to be male, history of comorbidities and non-Hispanic White, had higher waist circumference, AST, creatinine and HbA1c, and had lower albumin, education level, and income (Table 2).

**Table 1:** Baseline characteristics of all participants by the Log-dNLR quartile.

Variables	Total	Quartile 1	Quartile 2	Quartile 3	Quartile 4	*P* value
Age (years)	46.40(0.20)	44.88(0.30)	45.78(0.24)	46.39(0.27)	48.39(0.26)	< 0.0001
Sex, n (%)						0.02
Female	16489(48.63)	4093(21.85)	4133(25.34)	4213(26.54)	4050(26.28)	
Male	17903(51.37)	4538(23.46)	4407(25.98)	4423(25.59)	4535(24.97)	
Age group, n (%)						< 0.0001
20-39	11664(38.04)	3120(25.19)	2971(26.06)	2950(26.00)	2623(22.75)	
40-59	11476(38.71)	2885(21.64)	2990(26.40)	2868(26.19)	2733(25.78)	
60-79	9257(19.84)	2299(21.07)	2200(24.71)	2290(25.76)	2468(28.46)	
>80	1995(3.40)	327(15.74)	379(18.53)	528(26.80)	761(38.93)	
Education, n (%)						< 0.001
College and higher	17605(59.82)	4476(22.55)	4352(26.04)	4520(26.70)	4257(24.71)	
Middle and high school	12978(34.98)	3251(22.69)	3213(25.11)	3151(25.01)	3363(27.19)	
Primary school and less	3809(5.20)	904(24.03)	975(25.19)	965(25.57)	965(25.20)	
Race, n (%)						< 0.0001
Black	6823(10.16)	3009(44.63)	1516(22.28)	1218(17.85)	1080(15.23)	
Mexican	6083(8.04)	1264(21.20)	1636(27.48)	1668(27.17)	1515(24.15)	
Other	5700(11.93)	1459(24.93)	1559(27.88)	1390(24.50)	1292(22.70)	
White	15786(70.07)	2899(19.29)	3829(25.58)	4360(27.37)	4698(27.76)	
PIR, n (%)						0.04
<=1	6779(13.39)	1755(24.07)	1633(24.96)	1661(24.67)	1730(26.29)	
>1	27613(86.61)	6876(22.46)	6907(25.78)	6975(26.26)	6855(25.50)	
BMI (kg.m^2^)	28.75(0.07)	28.19(0.10)	28.52(0.10)	29.00(0.11)	29.21(0.12)	< 0.0001
Waist (cm)	98.53(0.18)	96.57(0.25)	97.81(0.26)	99.32(0.26)	100.19(0.27)	< 0.0001
HbA1c (%)	5.56(0.01)	5.55(0.01)	5.54(0.01)	5.55(0.01)	5.60(0.02)	< 0.001
Alt (IU/L)	26.02(0.15)	27..05(0.30)	26.07(0.23)	25.78(0.25)	25.30(0.41)	< 0.001
Ast (IU/L)	25.37(0.10)	26.33(0.21)	25.22(0.17)	24.95(0.16)	25.10(0.27)	< 0.0001
Albumin (g/dl)	4.30(0.00)	4.30(0.01)	4.32(0.01)	4.31(0.01)	4.29(0.01)	< 0.0001
Creatinine (mg/dl)	0.88(0.00)	0.88(0.00)	0.88(0.00)	0.88(0.00)	0.90(0.01)	0.002
Diabetes, n (%)						< 0.0001
No	25964(80.58)	6699(23.26)	6589(26.32)	6503(26.10)	6173(24.32)	
prediabetes	2610(7.15)	609(21.70)	615(23.47)	678(26.42)	708(28.40)	
Yes	5818(12.27)	1323(19.41)	1336(22.67)	1455(25.53)	1704(32.40)	
CVD, n (%)						< 0.0001
No	30780(92.00)	7903(22.95)	7779(26.04)	7731(26.08)	7367(24.93)	
Yes	3612(8.00)	728(19.47)	761(21.42)	905(25.74)	1218(33.37)	
Hypertension, n (%)						< 0.0001
No	20081(63.96)	5154(23.37)	5198(26.59)	5063(26.08)	4666(23.97)	
Yes	14311(36.04)	3477(21.44)	3342(24.04)	3573(26.01)	3919(28.51)	
Hyperlipidemia, n (%)						0.004
No	9571(29.03)	2569(24.00)	2396(26.22)	2281(25.04)	2325(24.75)	
Yes	24821(70.97)	6062(22.14)	6144(25.44)	6355(26.47)	6260(25.95)	
Smoke, n (%)						< 0.0001
No	18422(53.62)	4865(23.61)	4735(26.47)	4666(26.43)	4156(23.50)	
Yes	15970(46.38)	3766(21.86)	3805(24.75)	3970(25.62)	4429(28.04)	
Alcohol.user, n (%)						< 0.0001
Former	5835(13.81)	1403(21.60)	1389(23.97)	1426(26.14)	1617(28.29)	
Heavy	7081(21.99)	1701(22.39)	1746(24.56)	1810(25.86)	1824(27.19)	
Mild	11601(36.26)	2935(22.39)	2852(26.14)	2949(26.31)	2865(25.16)	
Moderate	5195(17.24)	1303(22.48)	1372(27.39)	1320(26.64)	1200(23.49)	
Never	4680(10.70)	1289(25.92)	1181(25.76)	1131(24.52)	1079(23.81)	
Stroke, n (%)						< 0.0001
No	3204(97.56)	8385(22.78)	8290(25.75)	9334(26.04)	8195(25.42)	
Yes	1188(2.44)	246(18.37)	250(22.34)	302(26.40)	390(32.89)	
Cancer, n (%)						< 0.0001
No	31496(91.53)	8044(23.02)	7903(25.93)	7885(25.89)	7664(25.17)	
Yes	2896(8.47)	587(19.00)	637(22.86)	751(27.80)	921(30.34)	
CKD, n (%)						< 0.0001
No	28359(86.64)	7430(23.40)	7219(26.14)	7163(26.13)	6547(24.30)	
Yes	6033(13.36)	1201(17.99)	1321(22.63)	1473(25.33)	2038(34.04)	
RA, n (%)						0.32
No	33533(98.14)	8415(22.66)	8346(25.73)	8425(26.04)	8347(25.57)	
Yes	859(1.86)	216(23.32)	194(22.33)	211(26.67)	238(27.68)	
Anemia, n (%)						< 0.0001
No	31634(94.60)	7857(22.49)	7935(25.89)	7983(26.16)	7859(25.47)	
Yes	2758(5.40)	774(26.00)	605(21.83)	653(24.22)	726(27.95)	
dNLR	1.52(0.01)	0.88(0.00)	1.24(0.00)	1.58(0.00)	2.32(0.01)	< 0.0001

**Notes:** All values represented are weighted means (SD), or counts (weighted percentage).

**Abbreviations:** SD, standard deviation; PIR, poverty index ratio; BMI, body mass index; ALT, alanine aminotransferase; AST, aspartate aminotransferase; CVD, cardiovascular disease; CKD, chronic kidney disease; RA, rheumatoid arthritis; Log-dNLR, Logarithm-transformed derived neutrophil-to-lymphocyte ratio.

**Table 2:** Baseline characteristics of all participants by survival status.

Variables	Total	Alive	Dead	*P* value
Age (years)	46.40(0.20)	44.31(0.20)	65.49(0.34)	< 0.0001
Sex, n (%)				< 0.0001
Female	16489(48.63)	14504(91.00)	1985(9.00)	
Male	17903(51.37)	14949(89.28)	2954(10.72)	
Age group, n (%)				< 0.0001
20-39	11664(38.04)	11468(98.21)	196(1.79)	
40-59	11476(38.71)	10700(94.08)	776(5.92)	
60-79	9257(19.84)	6647(75.82)	2610(24.18)	
>80	1995(3.40)	638(37.88)	1357(62.12)	
Education, n (%)				< 0.0001
College and higher	17605(59.82)	15886(93.25)	1719(6.75)	
Middle and high school	12978(34.98)	10766(86.80)	2212(13.20)	
Primary school and less	3809(5.20)	2801(76.46)	1008(23.54)	
Race, n (%)				< 0.0001
Black	6823(10.16)	5894(90.11)	929(9.89)	
Mexican	6083(8.04)	5423(95.23)	660(4.77)	
Other	5700(11.73)	5356(94.53)	344(5.47)	
White	15786(70.07)	12780(88.80)	3006(11.20)	
PIR, n (%)				< 0.001
<=1	6779(13.39)	5782(88.56)	997(11.44)	
>1	27613(86.61)	23671(90.36)	3942(9.64)	
BMI (kg.m^2^)	28.75(0.07)	28.74(0.07)	28.78(0.13)	0.81
Waist (cm)	98.53(0.18)	98.13(0.19)	102.15(0.30)	< 0.0001
HbA1c (%)	5.56(0.01)	5.52(0.01)	5.95(0.02)	< 0.0001
Alt (IU/L)	26.02(0.15)	26.04(0.13)	25.81(0.91)	0.80
Ast (IU/L)	25.37(0.10)	25.15(0.10)	27.39(0.48)	< 0.0001
Albumin (g/dl)	4.30(0.00)	4.32(0.00)	4.20(0.01)	< 0.0001
Creatinine (mg/dl)	0.88(0.00)	0.87(0.00)	1.02(0.01)	< 0.0001
Diabetes, n (%)				< 0.0001
No	25964(80.58)	23052(92.34)	2912(7.66)	
prediabetes	2610(7.15)	2147(86.93)	463(13.03)	
Yes	5818(12.27)	4254(77.42)	1564(22.58)	
CVD, n (%)				< 0.0001
No	30780(92.00)	27381(92.40)	3399(7.60)	
Yes	3612(8.00)	2072(63.89)	1540(36.11)	
Hypertension, n (%)				< 0.0001
No	20081(63.96)	18624(94.60)	1457(5.10)	
Yes	14311(36.04)	10829(81.64)	3482(18.36)	
Hyperlipidemia, n (%)				< 0.0001
No	9571(29.03)	8641(93.90)	930(6.10)	
Yes	24821(70.97)	20812(88.57)	4009(11.43)	
Smoke, n (%)				< 0.0001
No	18422(53.62)	16486(93.03)	1936(6.97)	
Yes	15970(46.38)	12967(86.76)	3003(13.24)	
Alcohol.user, n (%)				< 0.0001
Former	5835(13.81)	4189(77.91)	1646(22.09)	
Heavy	7081(21.99)	6552(94.62)	529(5.38)	
Mild	11601(36.26)	10044(90.98)	1557(5.79)	
Moderate	5195(17.24)	4765(94.21)	430(5.79)	
Never	4680(10.70)	3903(87.13)	777(12.87)	
Stroke, n (%)				< 0.0001
No	33204(97.56)	28783(90.83)	4421(9.17)	
Yes	1188(2.44)	670(61.89)	518(38.11)	
Cancer, n (%)				< 0.0001
No	31496(91.53)	27529(91.41)	3967(8.59)	
Yes	2896(8.47)	1924(76.22)	972(23.78)	
CKD, n (%)				< 0.0001
No	28359(86.64)	25718(93.32)	2641(6.68)	
Yes	6033(13.36)	3735(69.37)	2298(30.63)	
RA, n (%)				< 0.0001
No	33533(98.14)	28952(90.56)	4581(9.44)	
Yes	859(1.86)	501(66.65)	358(33.35)	
Anemia, n (%)				< 0.0001
No	31634(94.60)	27397(90.76)	4237(9.24)	
Yes	2758(5.40)	2056(78.94)	702(21.06)	
dNLR	1.52(0.01)	1.51(0.01)	1.68(0.02)	< 0.0001
Log-dNLR	0.39(0.00)	0.39(0.00)	0.41(0.00)	< 0.0001

**Notes:** All values represented are weighted means (SD), or counts (weighted percentage).

**Abbreviations:** SD, standard deviation; PIR, poverty index ratio; BMI, body mass index; ALT, alanine aminotransferase; AST, aspartate aminotransferase; CVD, cardiovascular disease; CKD, chronic kidney disease; RA, rheumatoid arthritis; Log-dNLR, Logarithm-transformed derived neutrophil-to-lymphocyte ratio.

### Association of dNLR with cardiovascular and all-cause mortality

In weighted univariable analysis, we incorporated all demographic, biochemical and clinical variables in our study, including age, age group, sex, education level, race, poverty index ratio, body mass index, waist circumference, HbA1c, Alt, Ast, albumin, serum creatinine, diabetes, cardiovascular disease, hypertension, hyperlipidemia, smoke, alcohol use, stroke, cancer status, CKD, RA, anemia, and Log-dNLR. Except for the variable of ALT, the other variables were significantly associated with all-cause mortality. Likewise, except for the variable of AST, the other variables also were associated with cardiovascular mortality. Log-dNLR was a potent risk factor for all-cause mortality (HR = 9.71, 95% CI: 6.61–14.28, *P* < 0.0001) and cardiovascular mortality (HR = 21.46, 95% CI: 11.52–39.97, *P* < 0.0001) (S1 and S2 Tables). Additionally, the cut-off value of dNLR for cardiovascular and all-cause mortality were 1.541 (95% CI: 0.640–0.582) and 1.628 (95% CI: 0.677–0.589) using the Youden index, the area under curve (AUC) were 0.627 and 0.594 respectively (S1 Fig).

The results of the multivariable Cox regressions for the association between Log-dNLR with cardiovascular and all-cause mortality are shown in Table 3 and Table 4. In all Cox models, the influence of Log-dNLR on cardiovascular and all-cause mortality was statistically significant. Among them, compared with quartile 1, participants in quartile 2, 3, and 4 were at higher risk of cardiovascular mortality in all models. Compared with quartile 1, participants in quartile 4 had 38% (HR = 1.38; 95% CI, 1.14–1.68) increased risk of cardiovascular mortality in model 3. Likewise, quartile 1 was the reference in all models, indicating that participants in quartile 4 were at a higher risk of all-cause mortality in all models. Specifically, participants in quartile 4 vs. those in quartile 1 demonstrated the risk of all-cause mortality increased by a factor of 16% (HR = 1.16; 95% CI, 1.06–1.27) respectively in model 3.

**Table 3:** Association of Log-dNLR with all-cause mortality, NHANES 1999-2018.

All-cause mortality	HR (95%CI), *P* Value							
	Crude model		Model 1		Model 2		Model 3	
**Q1**	1.00(ref)		1.00(ref)		1.00(ref)		1.00(ref)	
**Q2**	0.98(0.98,1.09)	0.76	0.97(0.87,1.07)	0.51	0.94(0.85,1.04)	0.26	0.94(0.85,1.04)	0.24
**Q3**	1.09(0.99,1.22)	0.09	1.02(0.92,1.14)	0.72	0.99(0.89,1.10)	0.86	0.96(0.86,1.07)	0.43
**Q4**	1.64(1.49,1.82)	<0.0001	1.32(1.21,1.44)	<0.0001	1.26(1.16,1.38)	<0.0001	1.16(1.06,1.27)	0.001
***P* for trend**		<0.0001		<0.0001		<0.0001		<0.0001

**Notes:** The Log-dNLR was converted from a continuous variable to a categorical variable. Data are presented as HR (95% CI). *P* value for trend was calculated by entering median values for each Log-dNLR quartile as a continuous variable in the Cox model. Crude model was adjusted no covariates. Model 1 was adjusted for age, sex, PIR, race, and education; Model 2 was adjusted for Model 1 + BMI, waist, albumin, AST, HbA1c (%), and serum creatinine. Model 3 was adjusted for Model 2 + DM status, CVD, hypertension, hyperlipidemia, cancer status, CKD, stroke, RA, anemia, alcohol use, and smoking status.

**Abbreviations:** NHANES: the National Health and Nutrition Examination Survey; PIR, poverty index ratio; BMI, body mass index; AST, aspartate aminotransferase; DM, diabetes; CVD, cardiovascular disease; CKD, chronic kidney disease; RA, rheumatoid arthritis; Log-dNLR, Logarithm-transformed derived neutrophil-to-lymphocyte ratio; HR, hazard ratio; CI, confidence interval; Ref, reference.

**Table 4:** Association of Log-dNLR with cardiovascular mortality, NHANES 1999-2018.

Cardiovascular mortality	HR(95%CI), *P* Value							
	Crude model		Model 1		Model 2		Model 3	
**Q1**	1.00(reference)		1.00(reference)		1.00(reference)		1.00(reference)	
**Q2**	1.18(0.95,1.46)	0.14	1.13(0.91,1.41)	0.27	1.09(0.87,1.36)	0.44	1.06(0.85,1.33)	0.64
**Q3**	1.26(1.02,1.56)	0.03	1.20(0.99,1.45)	0.07	1.13(0.92,1.38)	0.24	1.09(0.89,1.34)	0.41
**Q4**	2.12(1.73,2.59)	<0.0001	1.68(1.39,2.02)	<0.0001	1.63(1.34,1.98)	<0.0001	1.38(1.14,1.68)	0.001
***P* for trend**		<0.0001		<0.0001		<0.0001		<0.0001

**Notes:** The Log-dNLR was converted from a continuous variable to a categorical variable. Data are presented as HR (95% CI). *P* value for trend was calculated by entering median values for each Log-dNLR quartile as a continuous variable in the Cox model. Crude model was adjusted no covariates. Model 1 was adjusted for age, sex, PIR, race, and education; Model 2 was adjusted for Model 1 + BMI, waist, albumin, ALT, HbA1c (%), and serum creatinine. Model 3 was adjusted for Model 2 + DM status, CVD, hypertension, hyperlipidemia, cancer status, CKD, stroke, RA, anemia, alcohol use, and smoking status.

**Abbreviations:** NHANES: the National Health and Nutrition Examination Survey; PIR, poverty index ratio; BMI, body mass index; ALT, alanine aminotransferase; DM, diabetes; CVD, cardiovascular disease; CKD, chronic kidney disease; RA, rheumatoid arthritis; Log-dNLR, Logarithm-transformed derived neutrophil-to-lymphocyte ratio; HR, hazard ratio; CI, confidence interval; Ref, reference.

### RCS analysis

**Fig 2:**
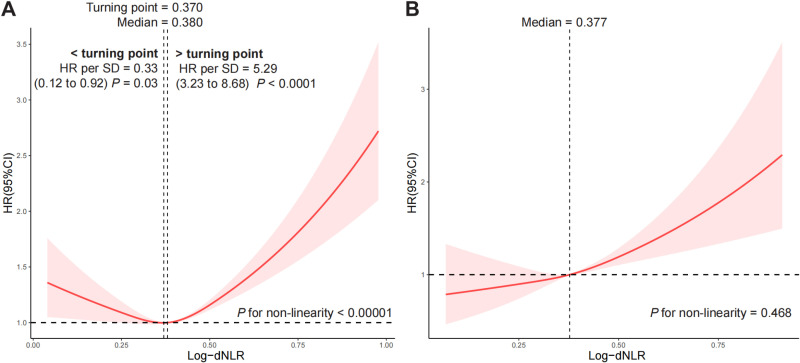
RCS analysis of the association between the Log-dNLR with all-cause and cardiovascular mortality. **Notes:** Vertical reference bars identified these inflection points and the median Log-dNLR. The red line and the shaded area symbolize the HR and corresponding 95% CI, respectively. (A) RCS curve of the association between Log-dNLR and all-cause mortality. The association was adjusted for age, sex, PIR, race, education BMI, waist, albumin, ALT, HbA1c (%), serum creatinine, DM status, CVD, hypertension, hyperlipidemia, cancer status, CKD, stroke, RA, anemia, alcohol use, and smoking status. (B) RCS curve of the association between Log-dNLR and cardiovascular mortality. The association was adjusted for age, sex, PIR, race, education BMI, waist, albumin, AST, HbA1c (%), serum creatinine, DM status, CVD, hypertension, hyperlipidemia, cancer status, CKD, stroke, RA, anemia, alcohol use, and smoking status. **Abbreviations:** NHANES: the National Health and Nutrition Examination Survey; PIR, poverty index ratio; BMI, body mass index; AST, aspartate aminotransferase; DM, diabetes; CVD, cardiovascular disease; CKD, chronic kidney disease; RA, rheumatoid arthritis; Log-dNLR, Logarithm-transformed derived neutrophil-to-lymphocyte ratio; HR, hazard ratio; CI, confidence interval; Ref, reference.

The associations between Log-dNLR with cardiovascular and all-cause mortality were further researched using the RCS curves. First, we found an inverted U relationship (*P* for nonlinear <  0.0001) between Log-dNLR and all-cause mortality (Fig 2A). The turning point occurred around Log-dNLR of 0.370, and the median number was 0.380. Regarding strong U-shaped relationship between Log-dNLR and all-cause mortality (*P* for nonlinear < 0.00001), Fig 2A showed a substantial, significant decline of the risk within lower range of Log-dNLR, which arrived the lowest risk in 0.370, and increased thereafter. Below 0.370, HR per SD higher Log-dNLR was 0.33 (HR = 0.33; 95% CI, 0.12–0.92, *P* = 0.03). Above 0.370, the HR per SD higher Log-dNLR was 5.29 (HR = 5.29; 95% CI, 3.23–8.68, *P* < 0.0001). Contrarily, Fig 2B revealed that positive linear correlation between Log-dNLR and cardiovascular mortality (*P* for nonlinear = 0.468). Results of the RCS analysis by age groups or DM status revealed that Log-dNLR was positively correlated with cardiovascular mortality in participants over 60 years old and participants with DM or without DM (S2 Fig). Similar to the relationships between the overall Log-dNLR and all-cause mortality, we also observed a nonlinear U relationship between Log-dNLR and all-cause mortality in male individuals and individuals with hypertension or without hypertension. A reversed L-shaped curves relationship between Log-dNLR and all-cause mortality was shown in female adults (S3 fig). Specific *P* for nonlinear and turning points are labeled on the figure.

### Subgroup analysis

To investigate further the relationship between Log-dNLR with cardiovascular and all-cause mortality among different populations, we carried out subgroup analysis based on multivariable Cox regression, stratified by sex, age group, race, education, DM status, CVD, smoking status, hyperlipidemia, hypertension, stroke, CKD, cancer status, RA, anemia, and alcohol use. The results of subgroup analysis showed that the association between Log-dNLR with cardiovascular and all-cause mortality among different populations (Table 5 and Table 6). The association remained robust between higher Log-dNLR and higher risks of cardiovascular and all-cause mortality in all subgroups. We calculated HR with 95% CI for participants in quartile 4 compared to quartile 1 in various subgroups and the *P* values of the interactions are shown in Table 5 and Table 6. For all-cause mortality, we found evidence of significant interaction by sex and hypertension (*P* for interaction = 0.025, 0.005, respectively). Interactions of Log-dNLR with age group and DM status on associations with cardiovascular mortality were significant (*P* for interaction = 0.007, 0.004, respectively).

**Table 5:** Subgroup analyses of the association between Log-dNLR and all-cause mortality.

Variables	Q1	Q2	Q3	Q4	*P* for trend	*P* for interaction
Age group						0.195
20-39	ref	1.211(0.785,1.869)	0.860(0.507,1.459)	1.380(0.865,2.203)	0.177	
40-59	ref	0.808(0.638,1.024)	0.840(0.641,1.100)	1.088(0.865,1.368)	0.470	
60-79	ref	0.972(0.844,1.120)	1.076(0.926,1.251)	**1.400(1.219,1.608)**	**<0.0001**	
>80	ref	1.125(0.915,1.382)	1.142(0.935,1.394)	**1.344(1.112,1.624)**	**0.001**	
Sex						**0.025**
Male	ref	0.939(0.808,1.091)	1.145(0.973,1.347)	**1.516(1.299,1.770)**	**<0.0001**	
Female	ref	1.013(0.855,1.201)	0.969(0.816,1.150)	**1.343(1.178,1.530)**	**<0.0001**	
Race						0.35
white	ref	0.966(0.842,1.107)	1.049(0.913,1.205)	**1.402(1.227,1.602)**	**<0.0001**	
mexican	ref	0.814(0.551,1.203)	0.936(0.652,1.344)	**1.393(1.007,1.927)**	**0.007**	
black	ref	1.128(0.919,1.384)	0.996(0.797,1.245)	**1.464(1.182,1.814)**	**0.004**	
other	ref	1.032(0.712,1.497)	1.380(0.913,2.088)	1.139(0.767,1.693)	0.35	
Education						0.87
college and higher	ref	1.017(0.836,1.236)	1.168(0.967,1.411)	**1.503(1.269,1.780)**	**<0.0001**	
middle and high school	ref	0.977(0.838,1.139)	1.067(0.912,1.249)	**1.468(1.300,1.657)**	**<0.0001**	
primary school and less	ref	0.898(0.690,1.169)	0.925(0.690,1.240)	**1.338(1.040,1.721)**	**0.009**	
Smoke						0.648
No	ref	0.964(0.805,1.155)	1.023(0.854,1.224)	**1.412(1.189,1.677)**	**<0.0001**	
Yes	ref	0.987(0.867,1.123)	1.13(0.978,1.305)	**1.472(1.286,1.684)**	**<0.0001**	
DM						0.27
No	ref	0.955(0.832,1.097)	1.075(0.940,1.228)	**1.501(1.310,1.719)**	**<0.0001**	
prediabetes	ref	1.068(0.747,1.525)	1.076(0.726,1.596)	**1.546(1.105,2.163)**	**0.007**	
Yes	ref	1.071(0.869,1.319)	**1.248(1.007,1.546)**	**1.457(1.195,1.777)**	**<0.0001**	
CVD						0.817
No	ref	0.944(0.826,1.078)	1.016(0.879,1.174)	**1.365(1.210,1.540)**	**<0.0001**	
Yes	ref	1.066(0.853,1.333)	1.181(0.956,1.458)	**1.554(1.285,1.878)**	**<0.0001**	
Stroke						0.843
No	ref	0.982(0.876,1.101)	1.078(0.954,1.218)	**1.473(1.318,1.647)**	**<0.0001**	
Yes	ref	0.848(0.582,1.234)	0.959(0.692,1.329)	1.265(0.873,1.834)	0.078	
Hypertension						**0.005**
No	ref	0.988(0.823,1.186)	0.879(0.701,1.101)	1.171(0.960,1.429)	0.156	
Yes	ref	1.017(0.894,1.158)	**1.232(1.096,1.384)**	**1.640(1.461,1.840)**	**<0.0001**	
Hyperlipidemia						0.881
No	ref	1.063(0.822,1.375)	1.107(0.834,1.470)	**1.453(1.105,1.910)**	**0.003**	
Yes	ref	0.958(0.850,1.080)	1.081(0.954,1.224)	**1.497(1.345,1.665)**	**<0.0001**	
Cancer						0.74
No	ref	0.973(0.868,1.091)	1.069(0.939,1.216)	**1.441(1.285,1.615)**	**<0.0001**	
Yes	ref	0.886(0.689,1.140)	0.952(0.752,1.203)	**1.301(1.073,1.578)**	**0.001**	
Alcohol.user						0.409
mild	ref	0.955(0.784,1.164)	1.109(0.907,1.356)	**1.525(1.249,1.862)**	**<0.0001**	
moderate	ref	1.002(0.716,1.403)	0.909(0.643,1.284)	**1.520(1.108,2.084)**	**0.006**	
never	ref	0.882(0.663,1.174)	1.141(0.907,1.356)	**1.525(1.249,1.862)**	**<0.0001**	
heavy	ref	0.990(0.692,1.417)	0.833(0.559,1.242)	1.175(0.833,1.659)	0.425	
former	ref	1.026(0.850,1.238)	1.233(1.001,1.518)	**1.508(1.236,1.840)**	**<0.0001**	
CKD						0.055
No	ref	0.898(0.783,1.029)	0.935(0.795,1.100)	**1.235(1.077,1.417)**	**<0.001**	
Yes	ref	1.124(0.966,1.308)	**1.265(1.110,1.441)**	**1.524(1.329,1.748)**	**<0.0001**	
RA						0.945
No	ref	0.970(0.865,1.088)	1.078(0.956,1.215)	**1.484(1.335,1.649)**	**<0.0001**	
Yes	ref	1.158(0.724,1.851)	1.246(0.771,2.012)	1.537(0.991,2.383)	0.055	
Anemia						0.233
No	ref	0.992(0.886,1.110)	1.060(0.936,1.201)	**1.486(1.331,1.659)**	**<0.0001**	
Yes	ref	0.985(0.747,1.297)	**1.326(1.029,1.709)**	**1.525(1.191,1.952)**	**<0.001**	

**Notes:** Data are presented as HR (95% CI). *P* value for trend was calculated by entering median values for each dNLR quartile as a continuous variable in the Cox model. Adjusted for age, sex, PIR, race, education BMI, waist, albumin, ALT, HbA1c (%), serum creatinine, DM status, CVD, hypertension, hyperlipidemia, cancer status, CKD, stroke, RA, anemia, alcohol use, and smoking status.

**Abbreviations:** PIR, poverty index ratio; BMI, body mass index; ALT, alanine aminotransferase; DM, diabetes; CVD, cardiovascular disease; CKD, chronic kidney disease; RA, rheumatoid arthritis; Log-dNLR, Logarithm-transformed derived neutrophil-to-lymphocyte ratio; HR, hazard ratio; CI, confidence interval; Ref, reference.

**Table 6:** Subgroup analyses of the association between Log-dNLR and cardiovascular mortality, NHANES 1999-2018.

Variables	Q1	Q2	Q3	Q4	*P* for trend	*P* for interaction
Age group						**0.007**
20-39	ref	**5.178(2.074,9.925)**	**3.681(1.735,7.812)**	1.458(0.508,4.184)	0.827	
40-59	ref	0.688(0.383,1.234)	0.787(0.464,1.334)	1.235(0.726,2.102)	0.234	
60-79	ref	1.167(0.878,1.552)	1.059(0.797,1.406)	**1.774(1.372,2.294)**	**<0.0001**	
>80	ref	1.189(0.807,1.751)	**1.492(1.016,2.192)**	**1.575(1.114,2.294)**	**0.005**	
Race						0.797
white	ref	1.060(0.814,1.380)	1.160(0.896,1.503)	**1.659(1.277,2.156)**	**<0.0001**	
mexican	ref	1.086(0.541,2.181)	1.281(0.649,2.528)	**2.180(1.115,4.265)**	**0.007**	
black	ref	1.315(0.918,1.882)	1.396(0.931,2.092)	**1.624(1.086,2.429)**	**0.014**	
other	ref	1.905(0.887,4.093)	1.178(0.477,2.908)	1.500(0.702,3.202)	0.688	
Sex						0.059
Male	ref	1.176(0.852,1.625)	**1.480(1.130,1.939)**	**1.975(1.467,2.659)**	**<0.0001**	
Female	ref	1.078(0.791,1.468)	0.841(0.605,1.170)	**1.487(1.143,1.934)**	**0.004**	
Education						0.413
college and higher	ref	0.944(0.630,1.414)	1.247(0.866,1.797)	**1.741(1.253,2.420)**	**<0.0001**	
middle and high school	ref	1.330(0.971,1.822)	1.231(0.910,1.666)	**1.891(1.438,2.487)**	**<0.0001**	
primary school and less	ref	1.005(0.601,1.681)	0.908(0.459,1.797)	1.251(0.744,2.105)	0.373	
Smoke						0.669
No	ref	1.150(0.849,1.558)	1.425(1.008,2.016)	**1.768(1.299,2.408)**	**<0.0001**	
Yes	ref	1.124(0.825,1.531)	1.040(0.765,1.413)	**1.704(1.306,2.224)**	**<0.0001**	
DM						**0.004**
No	ref	0.987(0.731,1.332)	1.078(0.813,1.429)	**1.888(1.453,2.453)**	**<0.0001**	
prediabetes	ref	1.789(0.806,3.974)	1.222(0.511,2.921)	1.878(0.916,3.847)	0.137	
Yes	ref	1.344(0.912,1.982)	**1.623(1.156,2.278)**	**1.550(1.114,2.157)**	**0.007**	
CVD						0.178
No	ref	0.964(0.695,1.339)	1.192(0.903,1.574)	**1.607(1.218,2.120)**	**<0.0001**	
Yes	ref	1.341(0.966,1.863)	1.095(0.774,1.548)	**1.723(1.233,2.408)**	**0.001**	
Stroke						0.466
No	ref	1.118(0.882,1.419)	1.213(0.964,1.526)	**1.735(1.375,2.189)**	**<0.0001**	
Yes	ref	1.140(0.583,2.227)	0.795(0.426,1.484)	1.591(0.869,2.916)	**0.095**	
Hypertension						0.261
No	ref	1.117(0.691,1.804)	0.886(0.553,1.418)	1.363(0.887,2.093)	0.197	
Yes	ref	1.182(0.916,1.523)	**1.375(1.089,1.736)**	**1.936(1.549,2.421)**	**<0.0001**	
Hyperlipidemia						0.891
No	ref	1.155(0.654,2.038)	1.514(0.809,2.835)	**1.745(1.019,2.988)**	**0.024**	
Yes	ref	1.128(0.896,1.420)	1.161(0.915,1.474)	**1.804(1.462,2.227)**	**<0.0001**	
Cancer						0.734
No	ref	1.055(0.840,1.325)	1.184(0.945,1.482)	**1.693(1.363,2.103)**	**<0.0001**	
Yes	ref	1.283(0.752,2.190)	1.016(0.552,1.868)	**1.781(1.099,2.886)**	**0.02**	
Alcohol.user						0.101
moderate	ref	1.135(0.512,2.515)	0.831(0.382,1.908)	1.219(0.531,2.802)	0.756	
mild	ref	0.939(0.616,1.431)	1.336(0.893,1.997)	**2.045(1.404,2.979)**	**<0.0001**	
heavy	ref	1.337(0.688,2.599)	0.774(0.395,1.515)	1.319(0.735,2.366)	0.623	
never	ref	0.784(0.486,1.263)	1.012(0.635,1.613)	**1.704(1.097,2.645)**	**0.004**	
former	ref	**1.544(1.066,2.235)**	**1.622(1.123,2.343)**	**1.870(1.285,2.721)**	**0.002**	
CKD						0.086
No	ref	0.985(0.729,1.332)	0.903(0.673,1.212)	**1.362(1.007,1.843)**	**0.037**	
Yes	ref	1.366(0.993,1.878)	**1.549(1.172,2.048)**	**1.892(1.497,2.391)**	**<0.0001**	
RA						0.549
No	ref	1.108(0.875,1.402)	1.176(0.927,1.491)	**1.759(1.433,2.159)**	**<0.0001**	
Yes	ref	1.239(0.565,2.716)	1.246(0.556,2.794)	1.754(0.794,3.875)	0.176	
Anemia						0.182
No	ref	1.146(0.910,1.444)	1.108(0.883,1.391)	**1.731(1.389,2.157)**	**<0.0001**	
Yes	ref	1.067(0.639,1.781)	**1.785(1.069,2.980)**	**2.099(1.338,3.293)**	**<0.0001**	

**Notes:** Data are presented as HR (95% CI). *P* value for trend was calculated by entering median values for each Log-dNLR quartile as a continuous variable in the Cox model. Adjusted for age, sex, PIR, race, education BMI, waist, albumin, AST, HbA1c (%), serum creatinine, DM status, CVD, hypertension, hyperlipidemia, cancer status, CKD, stroke, RA, anemia, alcohol use, and smoking status. **Abbreviations:** PIR, poverty index ratio; BMI, body mass index; AST, aspartate aminotransferase; DM, diabetes; CVD, cardiovascular disease; CKD, chronic kidney disease; RA, rheumatoid arthritis; Log-dNLR, Logarithm-transformed derived neutrophil-to-lymphocyte ratio; HR, hazard ratio; CI, confidence interval; Ref, reference.

### Sensitivity analysis

Sensitivity analyses were performed to test the robustness of the results, including excluding participants who had CVD or cancer at baseline, participants who died less than 2 years of the follow-up and participants with the shortest follow-up duration (NHANES 2017–2018). The results of all sensitivity analyses based on model 3 did not change substantially compared with those of the main analysis (Table 7).

**Table 7:** Sensitivity analysis between Log-dNLR with cardiovascular and all-cause mortality based on model 3.

Outcomes	Log-dNLR				*P* for trend
	Q1	Q2	Q3	Q4	
**Cardiovascular mortality**					
Removing participants with CVD or cancer at baseline	ref	0.97(0.69,1.34)	1.22(0.94,1.57)	1.50(1.14,1.98)	**<0.001**
*P*	ref	0.97	0.07	**0.004**	
NHANES 1999−2016	ref	1.07(0.86,1.34)	1.09(0.89,1.34)	1.35(1.11,1.64)	**<0.001**
*P*	ref	0.54	0.41	**<0.001**	
Removing participants who died within 2 years of the follow-up	ref	1.09(0.87,1.38)	1.10(0.88,1.37)	1.40(1.11,1.67)	**<0.0001**
*P*	ref	0.44	0.42	**0.003**	
**All-cause mortality**					
Removing participants with CVD or cancer at baseline	ref	0.95(0.83,1.09)	0.96(0.83,1.11)	1.17(1.02,1.33)	**0.01**
*P*	ref	0.46	0.59	**0.02**	
NHANES 1999−2016	ref	0.94(0.85,1.04)	0.95(0.86,1.06)	1.16(1.06,1.27)	**<0.0001**
*P*	ref	0.22	0.38	**0.002**	
Removing participants who died within 2 years of the follow-up	ref	0.92(0.83,1.03)	0.96(0.86,1.08)	1.14(1.03,1.26)	**<0.001**
*P*	ref	0.15	0.54	**0.001**	

**Notes:** Data are presented as HR (95% CI). *P* value for trend was calculated by entering median values for each Log-dNLR quartile as a continuous variable in the Cox model. Adjusted for age, sex, PIR, race, education BMI, waist, albumin, AST, HbA1c (%), serum creatinine, DM status, CVD, hypertension, hyperlipidemia, cancer status, CKD, stroke, RA, anemia, alcohol use, and smoking status in cardiovascular mortality and adjusted for age, sex, PIR, race, education BMI, waist, albumin, ALT, HbA1c (%), serum creatinine, DM status, CVD, hypertension, hyperlipidemia, cancer status, CKD, stroke, RA, anemia, alcohol use, and smoking status in all-cause mortality.

**Abbreviations:** NHANES: the National Health and Nutrition Examination Survey; PIR, poverty index ratio; BMI, body mass index; ALT, alanine aminotransferase; AST, aspartate aminotransferase; DM, diabetes; CVD, cardiovascular disease; CKD, chronic kidney disease; RA, rheumatoid arthritis; Log-dNLR, Logarithm-transformed derived neutrophil-to-lymphocyte ratio; HR, hazard ratio; CI, confidence interval; Ref, reference.

### Predictive accuracy of dNLR

**Fig 3:**
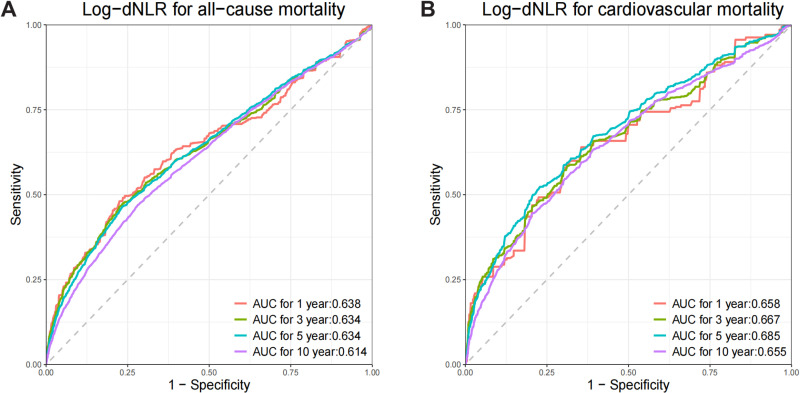
ROC curves of Log-dNLR in all-cause and cardiovascular mortality. **Notes**: (A) ROC curves of Log-dNLR in predicting 1-,3-, 5-, and 10-year all-cause mortality. (B) ROC curves of Log-dNLR in predicting 1-,3-, 5-, and 10-year cardiovascular mortality. **Abbreviations:** Log-dNLR, Logarithm-transformed derived neutrophil-to-lymphocyte ratio; ROC, receiver operating characteristics curve; AUC, area under the curve.

The effectiveness of Log-dNLR for mortality prediction was evaluated using weighted ROC curves. The AUC for all-cause mortality was 0.638 at 1 year, 0.634 at 3 years, 0.634 at 5 years, and 0.614 at 10 years (Fig 3A). For cardiovascular mortality, the AUC was higher as 0.658 at 1 year, 0.667 at 3 years, 0.685 at 5 years, and 0.655 at 10 years (Fig 3B). The findings suggested that the Log-dNLR exhibited moderate predictive power for in both the short- and long-term mortality.

## Discussion

For the first time, this large-scale cohort study evaluated the associations of dNLR with cardiovascular and all-cause mortality in 34,392 US participants based on NHANES (from 1999 to 2018). The results of our study confirmed that Log-dNLR was significantly positively associated with cardiovascular mortality and U relationship was showed in the association between Log-dNLR and all-cause mortality. This relationship persisted after adjusting for confounding factors. We also confirmed the robustness of our findings through multiple sensitivity analyses. Additionally, subgroup analysis revealed that the relationship between dNLR with cardiovascular and all-cause mortality varies across different populations. Participants with male or hypertension are at high risk for all-cause disease. Simultaneously, participants over 60 years of age or with DM have a higher risk of cardiovascular death.

dNLR as a novel inflammation marker that reflected the severity of systematic inflammation. Proctor et al. originally observed that dNLR has significant prognostic value in patients with cancer and could be universally applied in the risk stratification of patients undergoing chemotherapy [14]. Cancer-related inflammation promote tumor proliferation and invasion and has an adverse impact on outcomes of cancer patients [15]. Multiple clinical studies have confirmed that higher dNLR is associated with poor prognosis in cancer prognosis and cancer treatment [11,12,16,17]. Recently, researchers suggested that the mortality of acute coronary syndrome patients [10] and coronary heart disease patients [18] after percutaneous coronary intervention can be predicted through dNLR. Additionally, dNLR significantly predicted mortality of COVID-19 Omicron BA.2 infected patients [19] and COVID-19 patients [20] with solid tumors. dNLR has been widely incorporated into clinical settings. Despite cardiovascular disease remains the leading cause of death worldwide, the relationship dNLR with cardiovascular and all-cause mortality were not investigated in the current study.

The value of high dNLR depends on high levels of neutrophils or low levels of the Wbc count minus the neutrophil count. Denominator of dNLR is mostly composed of lymphocytes and monocytes. Neutrophils and Monocytes are key players in acute inflammation, and lymphocytes cell infiltration has a major role in the chronic inflammation. Neutrophils, lymphocytes and Monocytes are linked with cardiovascular disease progression and death by inflammation pathways. A large study from Denmark reported low levels of lymphocytes may be an early warning of future body diseases, while low lymphocyte count was associated with a higher risk of all-cause mortality [21]. Systematic reviews reported a strong correlation between the number of neutrophils and monocytes and the pathogenesis and progression of cardiovascular disease [5,6]. Hence, the combination of several leading indicators (neutrophils, monocyte, and lymphocyte) into a single composite metric could be more reflective of systemic inflammatory status. Particularly in diseased states neutrophils, monocyte, or lymphocyte counts are significantly lower or higher than normal values and in healthy states neutrophils, monocyte, or lymphocyte counts approximate the upper or lower limit of normal values.

In some cases, the peripheral monocyte count has been associated with detrimental prognosis for cancer patients [22]. Peripheral blood neutrophil counts or wbc counts are consistently associated with cardiovascular risk and outcome [23]. All analyses were adjusted for cardiovascular disease and cancer at baseline, but the status of cardiovascular disease and cancer at baseline may bring error to our results, and thus, participants with cardiovascular disease or cancer at baseline were excluded in sensitivity analysis. There are few notable changes as a result, indicating robustness of relationship between dNLR with cardiovascular and all-cause mortality. Our findings suggest that dNLR, a simple and readily available inflammatory biomarker, may serve as a useful tool for risk stratification in predicting cardiovascular and all-cause mortality in the general population. Given the significant role of systemic inflammation in cardiovascular disease progression and overall mortality, dNLR could be incorporated into existing risk assessment models to enhance prognostic accuracy. Additionally, since dNLR is derived from routine blood tests, it offers a cost-effective and practical approach for identifying individuals at higher mortality risk, particularly in resource-limited settings or primary care settings where advanced inflammatory markers may not be routinely available.

Due to different research directions and different subjects of study, the optimal cut-off value of dNLR may also be quite different. Lung immune prognostic index based on LDH and dNLR levels were proposed by a large multicenter study in France, among them, dNLR cut-off values were set to 3.0 [24]. Szkandera et al. defined a cutoff value of 2.3 for dNLR, high dNLR ≥ 2.3 seem to be associated with a poor clinical outcome in pancreatic cancer patients [17]. In the study that first proposed dNLR concept, the optimal threshold for dNLR to be 2.0 and associated with survival in cancer patients [14]. The published paper indicated that it concentrated in the range of 2.0 to 3.0. In this study, the optimal cut-off value of dNLR for cardiovascular and all-cause mortality was 1.541 and 1.628.

The ROC analysis demonstrated that Log-dNLR had a modest predictive ability for all-cause mortality. While this suggests that Log-dNLR alone is not a highly discriminative biomarker, it remains clinically relevant due to its ease of measurement and its established association with mortality risk. Previous studies have reported similar AUC values for other inflammatory markers, such as CRP and NLR, indicating that single inflammatory biomarkers generally have limited discriminatory power but still contribute to risk stratification in epidemiological settings. Moreover, it is important to recognize that AUC does not fully capture the clinical utility of a biomarker. Even biomarkers with modest AUC values may still be valuable when used in combination with clinical judgment and existing risk assessment tools. Future research should explore the integration of Log-dNLR into multivariable risk prediction models or its potential role in identifying high-risk subgroups where inflammation plays a more dominant role in mortality risk.

Although we grouped the participants by dNLR quartiles rather than optimal cut-off value, this has not impact on our results. Similar to previous studies, subgroup analysis and RCS analysis revealed that sex and hypertension status significantly affected the correlations between dNLR and all-cause mortality. One cross-sectional study found that isolated systolic hypertension is a high risk phenotype, leading to increased all-cause mortality and cardiovascular disease outcomes [25]. Similar findings were also reported in a prospective cohort study from China, hypertension can significantly increase the death risk of all-cause and cardiovascular disease, especially the highest risk of death in 35–59 years old group [26]. Although the pathophysiological relationship is unclear in the group-by-sex, evidence suggests that women typically live longer than men in the world [27]. This is also in accord with our observation that females have lower all-cause mortality rates than males. Simultaneously, age and DM status significantly affected the correlations between dNLR and cardiovascular mortality. Age and DM are the major risk factors for cardiovascular risk. A comparative study indicated that increased age is the largest driving factor for cardiovascular disease [28]. Diabetes mellitus is an important risk factor for cardiovascular mortality [29] and a first cardiovascular event and for worse outcomes after a cardiovascular event has occurred [30]. Again, our study demonstrated that participants who were over 60 years of age and who were DM had a higher risk of cardiovascular death.

The study has several advantages. A strength of our study is the association between dNLR with cardiovascular and all-cause mortality for the first time. We also identified special populations effects of dNLR on the cardiovascular and all-cause mortality. Importantly, this study was based on a stratified, multistage sampling method, had a large, nationally representative sample and could more effectively adjust for a variety of potential confounders. Second, our data is based on the NHANES database that used a stratified, multistage sampling method and extensive mortality follow-up, which increases the generalizability and reliability of our findings. A third advantage is that the time span from 1999–2018 years was wide enough and the follow-up period of 20 years was long enough to allow a sufficient number of incident cases of cardiovascular and all-cause mortality to be recorded. Last, various sensitivity analyses indicated the robustness of results and no meaningful differences emerged. Nevertheless, there are several limitations to this study. A first aspect is that the mortality data in NHANES data were based on death certificate data, which may lead to imprecise classification due to comorbidity and unforeseen events. Second, even though we controlled for potential confounders, the impact of residual confounding due to unknown or unmeasured confounders cannot exclude. Thirdly, the data collected via questionnaires might be susceptible to recall bias. Fourth, our present findings are confined to the American population; hence, they require validation in diverse demographic contexts. Fifth, although we have adjusted for several potential confounding variables, the existence of unmeasured confounders, such as the duration of the disease, drug dosage, and timing of administration, cannot be fully excluded.

## Conclusion

In conclusion, this cross-sectional study suggests a linear relationship between dNLR and cardiovascular mortality. Additionally, there is a U-shaped relationship between dNLR and all-cause mortality. In clinical practice, doctors should closely monitor the dNLR level and maintain at intermediate levels to prevent and appropriately reduce the cardiovascular and all-cause mortality risk.

## Supporting information

S1 Fig ROC curves of dNLR in cardiovascular (A) and all-cause (B) mortality. (TIF)

S2 Fig RCS analysis of the association between Log-dNLR and cardiovascular mortality among subgroups. Notes: Vertical reference bars identified these inflection points and the median Log-dNLR. The red line and the shaded area symbolize the HR and corresponding 95% CI, respectively. The association was adjusted for age, sex, PIR, race, education BMI, waist, albumin, AST, HbA1c(%), serum creatinine, DM status, CVD, hypertension, hyperlipidemia, cancer status, CKD, stroke, RA, anemia, alcohol use, and smoking status. (A-B) RCS curve of the association between Log-dNLR and cardiovascular mortality among no DM and DM participants; (C-F) RCS curve of the association between Log-dNLR and cardiovascular mortality among different age groups. Abbreviations: RCS, restricted cubic spline; NHANES, the National Health and Nutrition Examination Survey; PIR, poverty index ratio; BMI, body mass index; ALT, alanine aminotransferase; AST, aspartate aminotransferase; DM, diabetes; CVD, cardiovascular disease; CKD, chronic kidney disease; RA, rheumatoid arthritis; Log-dNLR, Logarithm-transformed derived neutrophil-to-lymphocyte ratio; HR, hazard ratio; CI, confidence interval.
(TIF)

S3 Fig RCS analysis of the association between Log-dNLR and all-cause mortality among subgroups. Notes: Vertical reference bars identified these inflection points and the median Log-dNLR. The red line and the shaded area symbolize the HR and corresponding 95% CI, respectively. The association was adjusted for age, sex, PIR, race, education BMI, waist, albumin, ALT, HbA1c(%), serum creatinine, DM status, CVD, hypertension, hyperlipidemia, cancer status, CKD, stroke, RA, anemia, alcohol use, and smoking status. (A-B) RCS curve of the association between Log-dNLR and all-cause mortality among female and male participants; (C-D) RCS curve of the association between Log-dNLR and all-cause mortality among no hypertension and hypertension participants. Abbreviations: RCS, restricted cubic spline; NHANES, the National Health and Nutrition Examination Survey; PIR, poverty index ratio; BMI, body mass index; ALT, alanine aminotransferase; AST, aspartate aminotransferase; DM, diabetes; CVD, cardiovascular disease; CKD, chronic kidney disease; RA, rheumatoid arthritis; Log-dNLR, Logarithm-transformed derived neutrophil-to-lymphocyte ratio; HR, hazard ratio; CI, confidence interval.
(TIF)

S1 Table Univariable Cox regression models of all-cause mortality. (XLSX)

S2 Table Univariable Cox regression models of cardiovascular mortality. (XLSX)
