# Supplementary figures and images for: Association of the derived neutrophil-to-lymphocyte ratio with cardiovascular and all-cause mortality
Source: PLoS One. 2025 Jun 5;20(6):e0324849. doi: 10.1371/journal.pone.0324849 (PMC12140284; doi:10.1371/journal.pone.0324849)

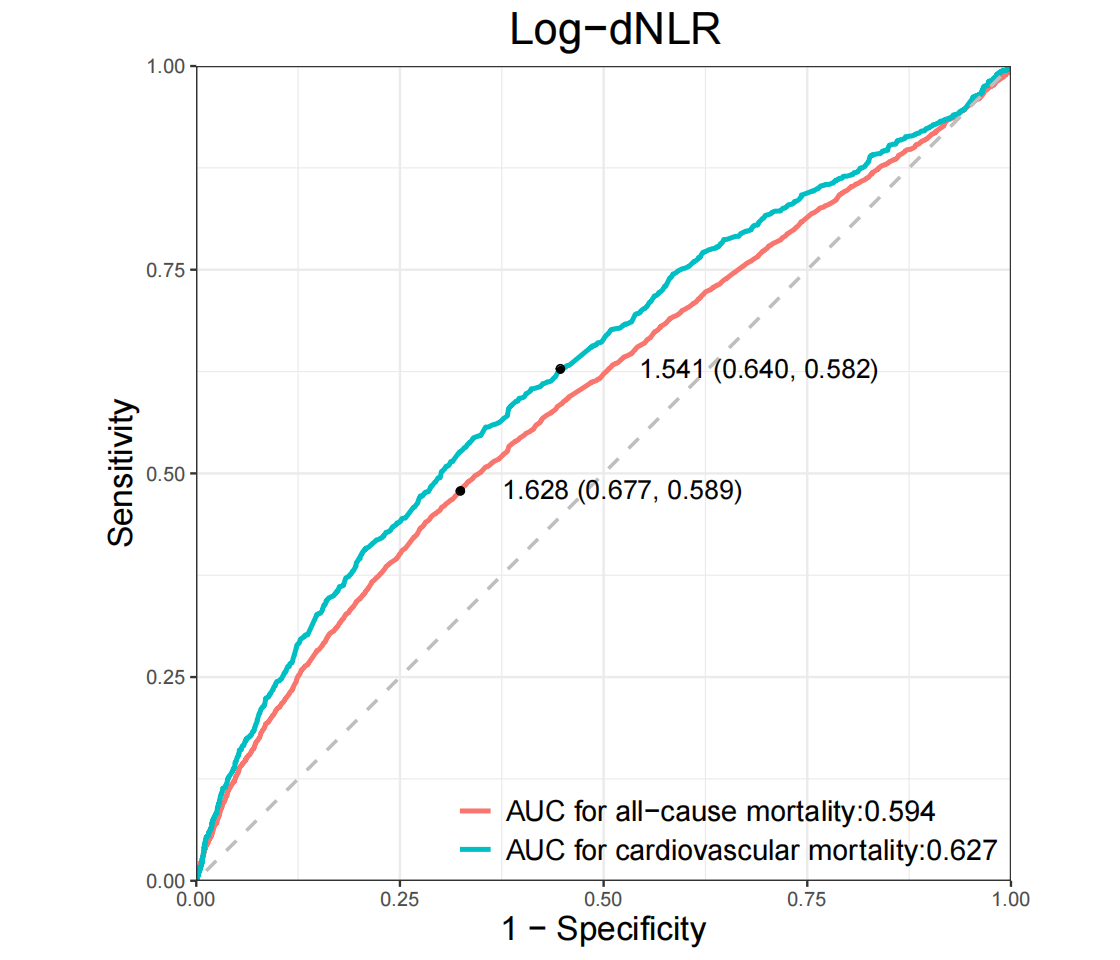

Supplement: S1 Fig — (TIF) [file pone.0324849.s001.tif]

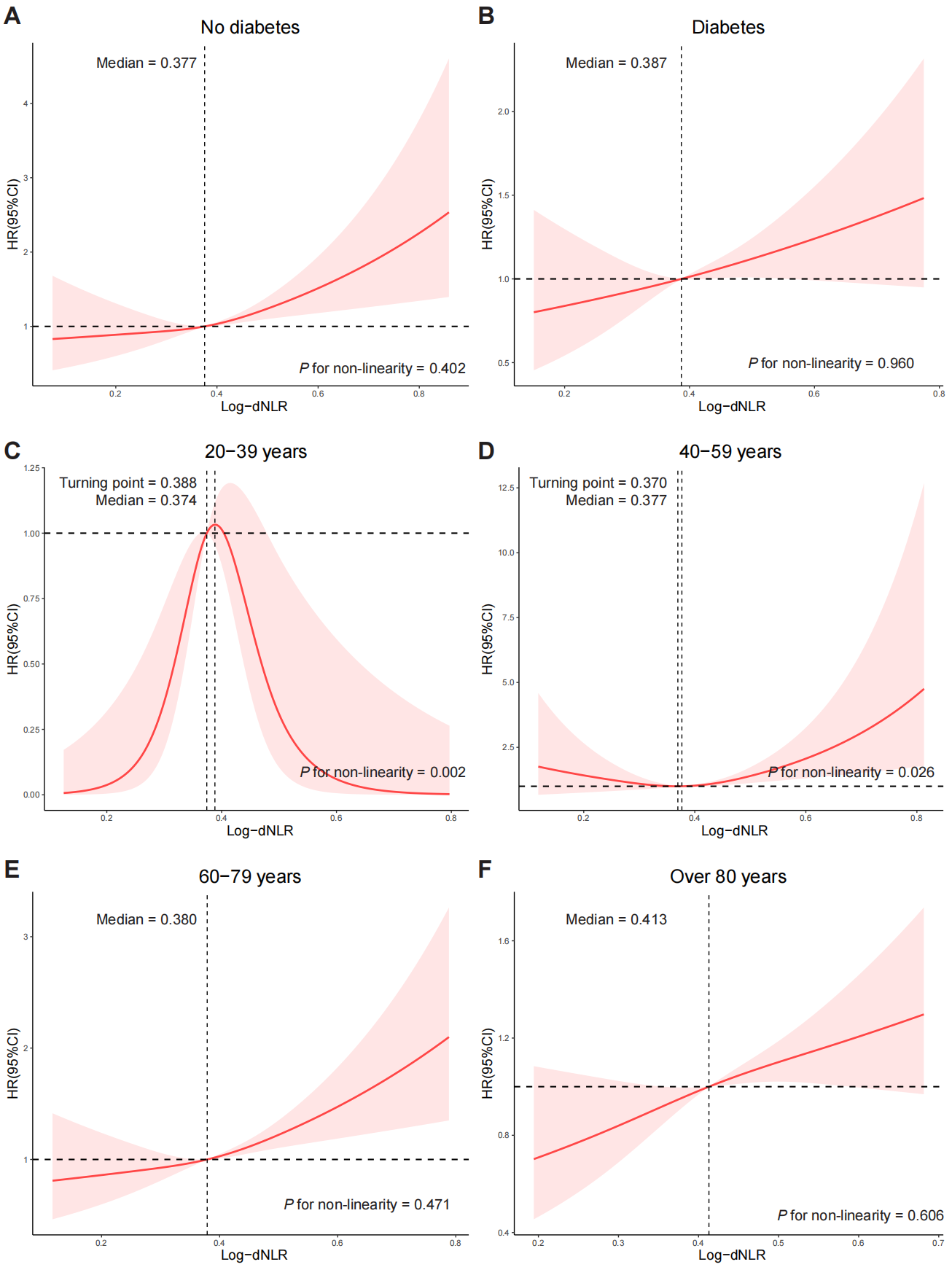

Supplement: S2 Fig — Notes: Vertical reference bars identified these inflection points and the median Log-dNLR. The red line and the shaded area symbolize the HR and corresponding 95% CI, respectively. The association was adjusted for age, sex, PIR, race, education BMI, waist, albumin, AST, HbA1c(%), serum creatinine, DM status, CVD, hypertension, hyperlipidemia, cancer status, CKD, stroke, RA, anemia, alcohol use, and smoking status. (A-B) RCS curve of the association between Log-dNLR and cardiovascular mortality among no DM and DM participants; (C-F) RCS curve of the association between Log-dNLR and cardiovascular mortality among different age groups. Abbreviations: RCS, restricted cubic spline; NHANES, the National Health and Nutrition Examination Survey; PIR, poverty index ratio; BMI, body mass index; ALT, alanine aminotransferase; AST, aspartate aminotransferase; DM, diabetes; CVD, cardiovascular disease; CKD, chronic kidney disease; RA, rheumatoid arthritis; Log-dNLR, Logarithm-transformed derived neutrophil-to-lymphocyte ratio; HR, hazard ratio; CI, confidence interval. (TIF) [file pone.0324849.s002.tif]

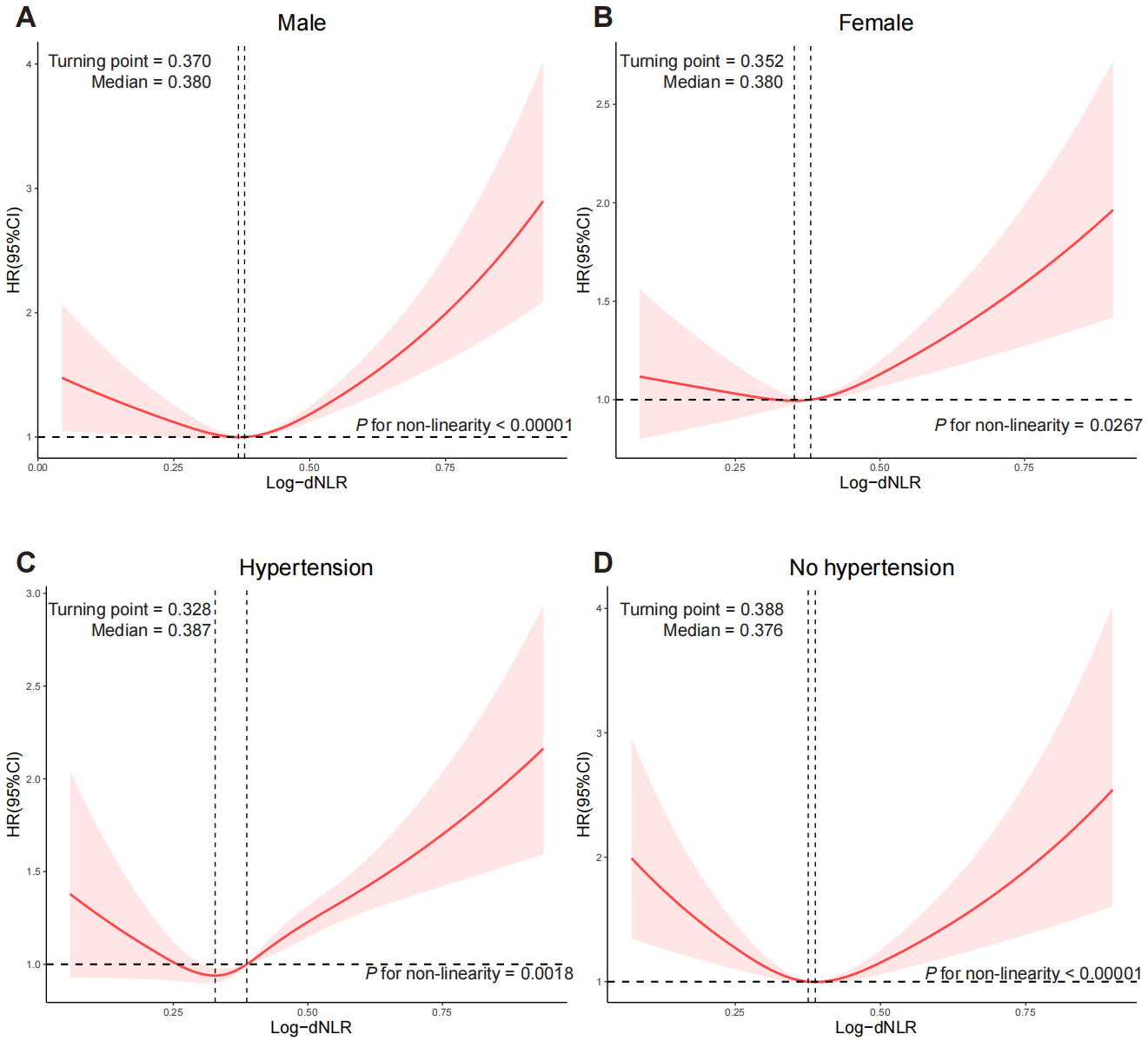

Supplement: S3 Fig — Notes: Vertical reference bars identified these inflection points and the median Log-dNLR. The red line and the shaded area symbolize the HR and corresponding 95% CI, respectively. The association was adjusted for age, sex, PIR, race, education BMI, waist, albumin, ALT, HbA1c(%), serum creatinine, DM status, CVD, hypertension, hyperlipidemia, cancer status, CKD, stroke, RA, anemia, alcohol use, and smoking status. (A-B) RCS curve of the association between Log-dNLR and all-cause mortality among female and male participants; (C-D) RCS curve of the association between Log-dNLR and all-cause mortality among no hypertension and hypertension participants. Abbreviations: RCS, restricted cubic spline; NHANES, the National Health and Nutrition Examination Survey; PIR, poverty index ratio; BMI, body mass index; ALT, alanine aminotransferase; AST, aspartate aminotransferase; DM, diabetes; CVD, cardiovascular disease; CKD, chronic kidney disease; RA, rheumatoid arthritis; Log-dNLR, Logarithm-transformed derived neutrophil-to-lymphocyte ratio; HR, hazard ratio; CI, confidence interval. (TIF) [file pone.0324849.s003.tif]
